# Gene copy number is associated with phytochemistry in *Cannabis sativa*

**DOI:** 10.1093/aobpla/plz074

**Published:** 2019-11-20

**Authors:** Daniela Vergara, Ezra L Huscher, Kyle G Keepers, Robert M Givens, Christian G Cizek, Anthony Torres, Reggie Gaudino, Nolan C Kane

**Affiliations:** 1 Kane Laboratory, Department of Ecology and Evolutionary Biology, University of Colorado Boulder, Boulder, CO, USA; 2 Steep Hill Inc., Berkeley, CA, USA

**Keywords:** Cannabinoid, CBD, chemotype, copy number variation, hemp, marijuana, metabolic pathway, THC

## Abstract

Gene copy number (CN) variation is known to be important in nearly every species where it has been examined. Alterations in gene CN may provide a fast way of acquiring diversity, allowing rapid adaptation under strong selective pressures, and may also be a key component of standing genetic variation within species. *Cannabis sativa* plants produce a distinguishing set of secondary metabolites, the cannabinoids, many of which have medicinal utility. Two major cannabinoids—THCA (delta-9-tetrahydrocannabinolic acid) and CBDA (cannabidiolic acid)—are products of a three-step biochemical pathway. Using whole-genome shotgun sequence data for 69 *Cannabis* cultivars from diverse lineages within the species, we found that genes encoding the synthases in this pathway vary in CN. Transcriptome sequence data show that the cannabinoid paralogs are differentially expressed among lineages within the species. We also found that CN partially explains variation in cannabinoid content levels among *Cannabis* plants. Our results demonstrate that biosynthetic genes found at multiple points in the pathway could be useful for breeding purposes, and suggest that natural and artificial selection have shaped CN variation. Truncations in specific paralogs are associated with lack of production of particular cannabinoids, showing how phytochemical diversity can evolve through a complex combination of processes.

## Teaser

The cannabinoid compounds, with high medicinal promise, are a main characteristic of the plant *Cannabis sativa*. These compounds were thought to have a simple mode of inheritance; however, our work describes multiple gene copies involved in their production. We found that first, varieties and individuals differ in the number of gene copies. Second, the number of copies may be related to the amount of cannabinoid production. Third, the expression of these genes also varies within varieties. An interesting remaining unknown from this study is the multiple other genes with unknown products, which could also have medicinal importance.

## Introduction

Gene copy number (CN) varies among individuals of the same species, which may have considerable phenotypic impacts (Stranger *et al.* 2007; Gaines *et al.* 2010). Both genome size and complexity can be increased by gene duplication (Losos *et al.* 2013), and new genes can be adaptive (Losos *et al.* 2013). Copy number variation seems to be related to gene function, with those encoding biochemical pathway hubs tending to have lower duplicability and evolution rates (Yamada and Bork 2009). The genes encoding for proteins that interact with the environment reportedly have a higher duplicability (Prachumwat and Li 2006; Yamada and Bork 2009), particularly, stress-response genes in multiple plant systems have a high mutation rate (Gaines *et al.* 2010; Hardigan *et al.* 2016). Therefore, CN variation can provide a path to rapid evolution in strong selective regimes (Gaines *et al.* 2010), such as changing environments (Żmieńko *et al.* 2014; Hardigan *et al.* 2016) or domestication (Swanson-Wagner *et al.* 2010; Ollivier *et al.* 2016).

Copy number variation occurs most commonly via gene duplication (Stranger *et al.* 2007; Losos *et al.* 2013) and CN variants are often selected during domestication (Swanson-Wagner *et al.* 2010; Ollivier *et al.* 2016). Three general modes of persistence of duplicated genes that may lead to CN variation have been proposed. The first mode is concerted evolution, in which the gene copies maintain similar sequence and function but the concentration of the gene product is augmented (Lynch 2007; Losos *et al.* 2013). The second mode is neofunctionalization in which a copied gene acquires a novel function (Lynch 2007; Losos *et al.* 2013). Finally, in subfunctionalization, the original function of the gene becomes split among the copies (Lynch 2007; Losos *et al.* 2013).

**Figure 1.**
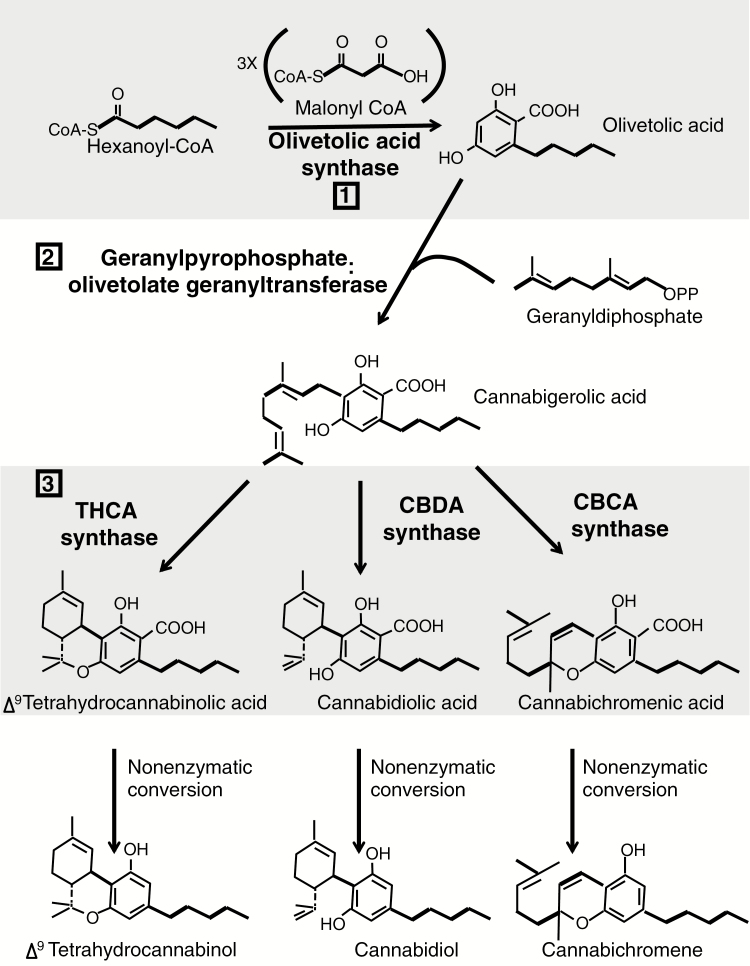
Cannabinoid synthesis pathway. The three-step biochemical pathway that produces the medically important cannabinoids in the trichomes of *C. sativa* flowers. Each enzymatic step is labelled with a number: 1) olivetolic acid synthase produces olivetolic acid; 2) olivetolate geranyltransferase produces CBGA; 3) THCA synthase, CBDA synthase and CBCA synthase produce THCA, CBDA or CBCA, respectively. The compounds are transformed to their neutral form (THC, CBD and CBC) with heat in a non-enzymatic conversion. Figure based on Page and Boubakir (2014).

Recently, humans have intensively bred for high levels of THCA (delta-9-tetrahydrocannabinolic acid) and CBDA (cannabidiolic acid) (ElSohly *et al.* 2000, 2016; ElSohly and Slade 2005; Volkow *et al.* 2014), the two most abundant and well-studied secondary metabolites (also referred to as specialized metabolites) produced by *Cannabis sativa*. As *Cannabis* has had a long history of domestication (Li 1973, 1974; Russo 2007), with recent intense selection (ElSohly *et al.* 2000, 2016; ElSohly and Slade 2005; Volkow *et al.* 2014), CN variation is likely to be found in these synthases (McKernan *et al.* 2015; Weiblen *et al.* 2015; Grassa *et al.* 2018; Laverty *et al.* 2019). *Cannabis sativa*, an angiosperm from the family Cannabaceae (Bell *et al.* 2010), produces numerous of these secondary metabolites called cannabinoids, which are a primary distinguishing characteristic of the plant. These two compounds—THCA and CBDA—when heated are converted to the neutral forms delta-9 tetrahydrocannabinol (THC) and cannabidiol (CBD), respectively (Russo 2011), which are the forms that interact with the human body (Hart *et al.* 2001). These compounds have a plethora of both long-known and recently discovered medicinal (Russo 2011; Swift *et al.* 2013; Volkow *et al.* 2014) and psychoactive properties (ElSohly and Slade 2005) and are most abundant in the trichomes of female flowers (Sirikantaramas *et al.* 2005; Gagne *et al.* 2012). The enzymes responsible for their production, THCA and CBDA synthases (hence THCAS and CBDAS), are alternative end catalysts of a biochemical synthesis pathway (Fig. 1; (Sirikantaramas *et al.* 2005; Gagne *et al.* 2012; Page and Boubakir 2014). Finally, certain *Cannabis* chemovars contain higher THCA concentrations (e.g. ‘marijuana-type’ cultivars), while other *Cannabis* chemovars contain higher CBDA concentrations (e.g. hemp and high-CBDA ‘marijuana’ varieties) (de Meijer *et al.* 1992; Rustichelli *et al.* 1998; Mechtler *et al.* 2004; Datwyler and Weiblen 2006). However, recent research has found genetic support for *Cannabis* phylogenetics that correlates strongly with leaf morphology (Clarke and Merlin 2013; Lynch *et al.* 2016), with the Broad Leaf Marijuana-type and the Narrow Leaf Marijuana-type used medically and recreationally, and the hemp group used for industrial purposes. The designations ‘Broad Leaf’ and ‘Narrow Leaf’ used in Lynch *et al.* (2016), and adopted in this study, imply that these groupings are based on leaf morphology. However, it is important to note that although leaf morphology serves as a useful designation, their grouping is based on molecular phylogenetics, not morphological.

It was once thought that THCAS and CBDAS were two Mendelian-inherited alleles from the same gene and that allelic variation determined the predominant cannabinoid composition (de Meijer *et al.* 1992, 2003; Hillig and Mahlberg 2004; Pacifico *et al.* 2006; Onofri *et al.* 2015). However, it has recently been established that there are multiple genes in close proximity that are responsible for the production of cannabinoids (McKernan *et al.* 2015; Weiblen *et al.* 2015; Grassa *et al.* 2018; McKernan *et al.* 2018; Laverty *et al.* 2019). Therefore, an alternative explanation for observed phytochemical diversity is that CN variation may contribute to different cannabinoid phenotypes in the *C. sativa* cultivars (McKernan *et al.* 2015).

Due to the medical potential of this pathway in regard to using cannabinoids for treating illnesses, and the possibility that CN variation in the genes that encode their enzymes may affect cannabinoid content, we explored the inter- and intra-cultivar differences in these genes. Since the discovery of the multiple paralogs of the cannabinoid synthases a new question arises of how CN variation in r of these paralogs relates to the chemotypes of the plants that contain them. Using two *de novo C. sativa* genome assemblies and an additional 67 Whole Genome Shotgun data sets from a diversity of cultivars, we addressed three questions: (i) Do lineages differ in number of cannabinoid synthase paralogs? (ii) Does cannabinoid content correlate to the number of respective synthase paralogs by cultivar? (iii) Do cannabinoid synthase paralogs vary in expression level by tissue and cultivar?

## Materials and Methods

### Genome assemblies and gene annotation within the assemblies

We used two different genome assemblies for this study. The first was from a high-THCA marijuana-type male, Pineapple Banana Bubba Kush (PBBK), sequenced using PacBio Single-Molecule Real-Time (SMRT) Long-Read (LR) technology (Eid *et al.* 2009; Rhoads and Au 2015), provided by Steep Hill, Inc. (NCBI GenBank Whole Genome Shotgun accession number MXBD01000000). Even though this is a male assembly, we believe that the genomic regions related to cannabinoid production are independent of the plant’s sex. However, further studies may elucidate the expression differences between male and female *Cannabis* plants that make females more prone to produce more cannabinoids in their flowers. The second assembly was constructed in 2011 from a high-THCA dioecious female marijuana-type Purple Kush (PK) plant, sequenced on the Illumina platform (van Bakel *et al.* 2011). Most results from this assembly will be given in the **Supporting Information—Methods and Results**. Both assemblies vary in their completeness, as each have some missing BLAST (Altschul *et al.* 1990; Gish and States 1993) hits as described below and in the **Supporting Information—Methods and Results**. Each assembly has some duplicated regions, with patterns of coverage suggesting that allelic variation at heterozygous loci lead to two different sequences assembled at a single genomic location. Because both are flawed due to these and other likely misassemblies (Vergara *et al.* 2016), it was necessary to use both assemblies, which allowed us to find at least one hit for every target gene in order to understand the whole cannabinoid pathway.

We found 11 and 5 BLAST hits for putative CBDA/THCA synthase genes in the PBBK and PK assembly, respectively, for a total of 16 potential paralogs in the CBDAS/THCAS gene family **[see** **Supporting Information—Table S1****]**. Based on percent-identity scores, we found a hit in each assembly that appears to code for THCAS. We identified two hits in the PBBK and one in the PK assemblies that likely code for CBDAS. We used the CBDAS and THCAS cDNA sequences as references with NCBI accession numbers AB292682.1 and JQ437488.1, respectively. We also found one hit in the PBBK assembly to the gene producing the third product variant of this pathway, cannabichromenic acid (CBCA) using a cDNA sequence as a reference (Page and Stout 2017).

**Figure 2.**
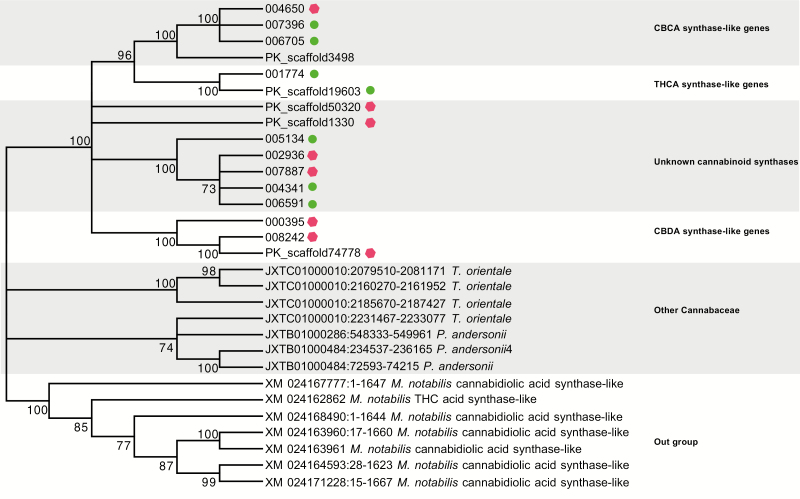
Maximum likelihood (ML) gene tree with paralogs from the CBDA/THCA synthase family. Relationship between 16 paralogs (11 from the PBBK assembly (prefix ‘00’) and five from the PK assembly (prefix ‘PK_scaffold’)). Green circles indicate full-length reading frames, red hexagons indicate truncated reading frames with homology to reference proteins extending beyond stop codons located within them. Paralogs are indicated to be CBDAS-like, THCAS-like or CBCAS-like. Many of the homologs have unknown function. Also included are two other species from the family Cannabaceae, *Parasponia andersonii* and *Trema orientale* with three and four sequences, respectively. The outgroup are sequences from the closely related species from the family Moraceae *Morus notabilis.* The numbers in the branches indicate the percent of 500 bootstraps that supported this topology. NCBI accession numbers for each of the proteins listed in the tree. All nucleotide data found on the Dryad repository (https://datadryad.org/stash/share/MsyF2os_zaKN6d9uoDLroX7O0RrW8kT8sPzep7WffLU).

We constructed a maximum likelihood (ML) tree using the default parameters in MEGA version 7 (Kumar *et al.* 2016) with the 16 CBDA/THCA synthase gene family from both assemblies to understand the relationships between them (Fig. 2). In order to discern the relationship between the CBDA/THCA synthase gene family, we identified putative homologs of CBDAS/THCAS in closely related species using a tblastx search against NCBI’s non-redundant database. We chose tblastx in lieu of blastx because it allows comparison of nucleotide sequences without the knowledge of any protein translation (Wheeler and Bhagwat 2007). We included 14 sequences from three species from the order Rosales, two of them also from the family Cannabaceae—*Trema orientale* and *Parasponia andersonii* with four and three sequences, respectively—and a more distantly related species from the family Moraceae as an outgroup, *Morus notabilis*, with seven sequences. Therefore, our ML tree included a total of 30 putative CBDAS/THCAS homolog sequences, 16 from *Cannabis*, 7 from two other species in the Cannabaceae and 7 from the outgroup *Morus.* All sequences are deposited on Dryad digital repository (https://datadryad.org/stash/share/MsyF2os_zaKN6d9uoDLroX7O0RrW8kT8sPzep7WffLU).

Finally, for the 16 sequences we found in the PBBK and PK assemblies, we calculated genetic distance and nucleotide composition using MEGA, and compared the non-synonymous to synonymous sites ratio between sequences with SNAP (Korber 2000).

### Genomic sequences, alignment and depth of coverage calculation

We used 67 Illumina platform whole-genome shotgun sequence libraries available from various *Cannabis* cultivars **[see** **Supporting Information—Table S2****]** from three major lineages within *C. sativa* (FLOCK; Duchesne and Turgeon 2012): 15 individuals from Broad Leaf Marijuana-type (broad-leaf), 31 from the Narrow Leaf Marijuana-type (narrow-leaf), 16 hemps and five unassigned individuals (Lynch *et al.* 2016). These groupings based on leaf morphology have been previously established (Clarke and Merlin 2013) and corroborated with genomic analyses (Lynch *et al.* 2016; Vergara *et al.* 2016). The 67 whole genomes used in this analysis have raw read lengths from 100 to 151 bp. The relationship between these 67 individuals has already been established and they have been assigned to these three lineages (broadly classified as broad-leaf, narrow-leaf and hemp). The classification of the drug-type lineages correlates strongly with leaf morphology, although it is important to note that the relationships were inferred based on genetic relatedness, rather than morphological characters. For detailed information on sequencing and the library prep these 67 genomes refer to Lynch *et al.* (2016).

We aligned the 67 libraries to both assemblies using Burrows-Wheeler alignment (BWA) version 0.7.10-r789 (Li and Durbin 2009), then calculated the depth of coverage using SAMtools version 1.3.1-36-g613501f (Li *et al.* 2009). The expected coverage at single copy sites was calculated with the aligned data divided by the genome size **[see** **Supporting Information—Table S2****]**, estimated to be 843 Mb for male and 818 Mb for female *Cannabis* plants (Sakamoto *et al.* 1998). Intrinsic similarity among paralogous genes—and thus probability that reads from different loci align to the same paralog—precluded establishing specific SNPs. However, we calculated the number of possible gene paralogs encoding each enzyme in the cannabinoid pathway (Fig. 1) for each cultivar using coverage from both assemblies. The estimated CN for each cannabinoid sequence was calculated as the average depth across that sequence divided by the expected coverage. This scaled depth was therefore used as a measure of gene CN for each cultivar.

To determine the highest total number of genes per cultivar for CBDAS/THCAS, the depth of coverage was calculated for each library when aligned to the PBBK assembly that had been modified to include only one paralog (PBBK scaffold 001774).

### Gene CN statistics

Differences in the estimated gene CN between the cultivars for each of the 16 in the CBDAS/THCAS gene family were determined using one-way ANOVAs on the CN of each gene as a function of the lineages (narrow-leaf, broad-leaf, hemp), with a later *post hoc* analysis to establish one-to-one group differences. Three ANOVAs were also performed for each of the lineages to determine within-group variation. The cultivars were then compared with either an ANOVA for cultivars with more than two samples (Carmagnola and Afghan Kush) or a paired *t*-test for those with two individuals (Chocolope, Kompolti, Feral Nebraska, Durban Poison and OG Kush; **see** **Supporting Information—Methods and Results**). Additionally, we performed a Phylogenetic Generalized Least Squares (PGLS) model with the package NLME (Pinheiro *et al.* 2019) on the R statistical platform (R Core Team 2013) to determine possible correlations between the depths of each paralog correcting for relatedness between cultivars.

### Phenotypic analysis

#### Chemotypes.

Cannabinoid concentration profiles (chemotypes) were generated by Steep Hill, Inc. following their published protocol (Lynch *et al.* 2016). Briefly, data collection was performed using high-performance liquid chromatography (HPLC) with Agilent (1260 Infinity, Santa Clara, CA, USA) and Shimadzu (Prominence HPLC, Columbia, MD, USA) equipment with 400–6000 mg of sample. We report the estimated total cannabinoid content calculated from the acidic and neutral form of each cannabinoid as in Vergara *et al.* (2017) and used these values to obtain chemotypic averages for each cultivar. We had the specific chemotypes for eight cultivars which also were sequenced. In these cases, we used individual values instead of the averages **[see** **Supporting Information—Table S3****]**.

#### ***CN vs***.***chemotype correlation***.

To evaluate the relationship between the estimated gene CN for each of the genes and chemotype, we performed PGLS correlations between the chemotype and the average estimated gene CN per gene **[see** **Supporting Information—Methods and Results****]** while correcting for phylogenetic relatedness. Only cultivars with matching data in the genomic analysis were analysed, for a total of 35 individuals from 22 different cultivars. The broad-leaf group had 10 individuals from six cultivars, the narrow-leaf had 15 individuals from 13 cultivars, the hemp group had six individuals from one cultivar, and there were four individuals from three cultivars that were not assigned to any group (Lynch *et al.* 2016). The chemotype data represent 822 individuals from 22 unique cultivars. Some caveats of this analysis are that we averaged the chemotypes for most of the shared cultivars except for the eight cultivars for which we had the specific chemotype for that particular genotype **[see** **Supporting Information—Table S3****]**. Additionally, since *Cannabis* cultivars are notoriously mislabelled (Sawler *et al.* 2015; Vergara *et al.* 2016), some of the values that are part of the averages could be ambiguous. However, an important strength of this average is that effects of environmental variation and statistical noise are minimized, improving our ability to assess genetically based variation. We also performed PGLS correlations to the sum of all cannabinoids to examine whether CN variation had an effect on overall cannabinoid content.

### Expression analysis

As a proxy measure of differential expression of the genes on the cannabinoid pathway, we aligned three published RNA sequences derived, respectively, from the flower and root of PK and the flower of the hemp cultivar Finola (van Bakel *et al.* 2011) to the whole PBBK assembly. We used the Tuxedo suite, which includes Bowtie2 v2.3.4.1 (Langmead and Salzberg 2012) for RNA alignment, TopHat for mapping v2.1.1 (Trapnell *et al.* 2009) and Cufflinks v2.2.1 for assembling transcripts and testing for differential expression (Trapnell *et al.* 2010). We used CummeRbund’s output from the RNA-Seq results (Trapnell *et al.* 2012).

## Results

### CBDA/THCA synthase family

The quantification of relatedness between the combined 16 CBDA/THCA synthase paralogs drawn from both genome assemblies revealed distinct clusters (Fig. 2). Two paralogs, located on contig 001774 and PK scaffold 19603, from the PBBK and PK assemblies, respectively, cluster together with 100 % bootstrap support and are related to genes known to be involved in THCA production. Similarly, the paralogs we infer to be CBDA synthases—two from the PBBK assembly (000395 and 008242) and one from the PK assembly (74778)—also cluster together. We found a cluster of four genes, three from the PBBK assembly and one from the PK assembly, that we infer to be CBCA synthases. All genes used from the two other Cannabacea species *T. orientale* and *P. andersonii* cluster together. Similarly, the genes from the outgroup *M. notabilis* also form a cluster, excluding the 16 *Cannabis* sequences.

### Gene CN statistics

The one-way ANOVAs for each gene and *post hoc* analysis show that the CN of some of the paralogs differs among the three major cultivar groups (**see** **Supporting Information—Table S4**—between-group comparison). However, the *post hoc* analysis with the medians from the broad-leaf, narrow-leaf and hemp groups shows that hemp lineage differs from the other two groups in paralog CN, independent of which assembly was used as a reference.

**Figure 3.**
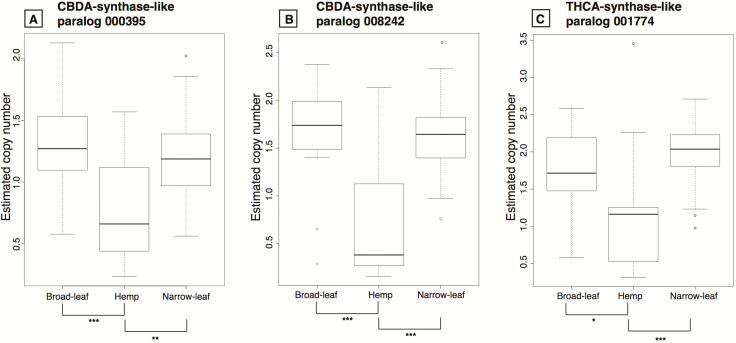
Estimated CN by group for three of the CBDAS/THCAS paralogs. Box plots for three of the paralogs from the 11 total paralogs of the CBDA/THCA synthase family from the PBBK assembly. Panels (A) and (B) depict the CBDAS-like genes and panel (C) is the THCAS-like gene. Significant values between the comparisons are given in the horizontal bars below each panel: ***P < 0.001, **P < 0.003, *P < 0.03. The estimated CN by group from the two CBDAS/THCAS paralogs in the PK assembly is given in **Supporting Information—Fig. S1**.

Hemp appears to differ the most from the other two lineages in the CN of the three CBDAS-like and the two THCAS-like paralogs both between and within lineages (Fig. 3), because for the three paralogs, the hemp lineage has the lowest mean **[see** **Supporting Information—Table S4****]** and median (Fig. 3) CN.

### Phenotypic analysis

#### CN vs. chemotype correlation.

**Figure 4.**
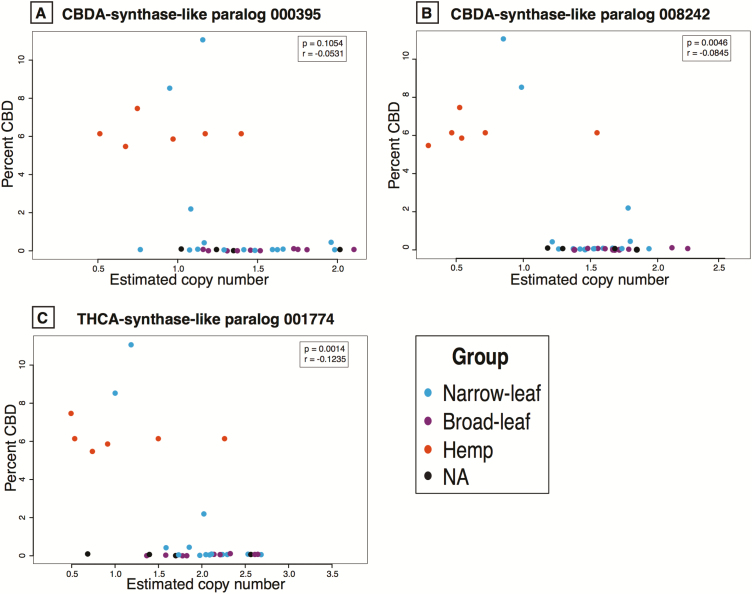
Correlations between the percent CBDA and the estimated CN for the three CBDA/THCA synthase paralogs. Two CBDAS-like genes (panels A and B) and one THCAS-like gene (panel C) correlated to CBDA production. All correlations are negative and those shown in (B) and (C) are significant. Correlation coefficient and *P*-values in the inset after correction for relatedness. All correlation values between all genes and all cannabinoids are given in **Supporting Information—Tables S5 and S6**, respectively.

**Figure 5.**
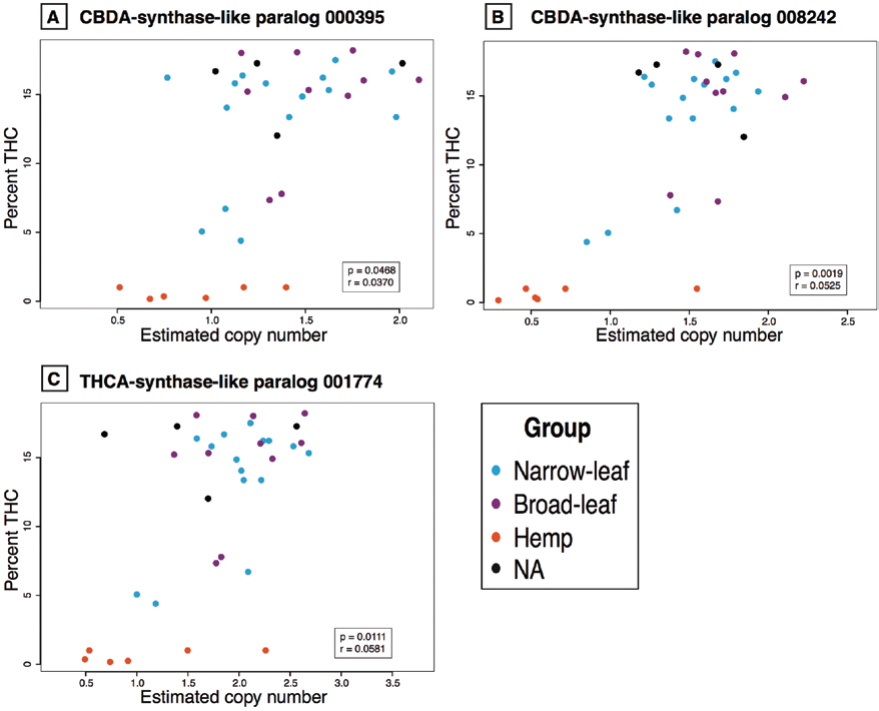
Correlations between the percent THCA and the estimated CN for three CBDA/THCA synthase paralogs. The two CBDAS-like genes (panels A and B) and the one THCAS-like gene (panel C) are positively and significantly correlated at the *P* < 0.05 level to the percent THCA. Correlation coefficient and *P*-values in the inset after correction for relatedness. All correlation values between all genes and all cannabinoids are given in **Supporting Information—Tables S5 and S6**, respectively.

After correcting for relatedness, most correlations between the cannabinoid levels and the synthase gene CN lack significance both in the modified and original assemblies **[see** **Supporting Information—Table S5****]**. However, the original assemblies had important significant correlations before correcting for relatedness **[see** **Supporting Information—Table S5****]**. For CBD chemotypic abundance (after correcting for relatedness) CNs of one (008242) of the two CBDAS-like paralogs significantly but negatively correlate (Fig. 4A and B). Interestingly, the THCAS-like paralog 001774 is also negatively but significantly correlated to CBD accumulation (Fig. 4C). For THC chemotypic abundance after correcting for relatedness, all CBDAS/THCAS paralog CNs show significant positive correlations (Fig. 5). All other correlations between chemotypic abundance and the multiple gene CNs are given in **Supporting Information—Table S5**. The PGLS correlations to the sum of all cannabinoids show similar patterns as the correlations to single cannabinoids. The patterns shown in Figs 4 and 5 are similar to the ones observed when using the PK genome as a reference (**see** **Supporting Information—Fig. S2A and B** for correlations with percent CBD and **Supporting Information—Fig. S2C and D** for correlations with percent THC).

We found that paralog 006705 had the highest BLAST percent-identity score (99.93 %) to the cDNA from the CBCA synthase. Additionally, the two other paralogs that cluster in the same group (007396 and 004650; Fig. 1) also show a high-percent identity (99.87 and 99.81 %, respectively) to CBCA synthase. None of the 16 CBDA/THCA synthase-family paralogs correlate with the accumulation of CBC **[see** **Supporting Information—Table S5****]** after correcting for relatedness. Additionally, the PGLS model with paralogs 007396, 004650 and 006705 did not show any significance. However, three different paralogs (50320, 002936 and 007887) with lower BLAST scores showed a significant correlation with CBC accumulation before correcting for relatedness.

### Expression analysis

Our proxy expression analysis suggests differences in the gene products between cultivars and tissues (Table 1). Even though the differences are not significant, the marijuana-type cultivar PK seems to express the olivetolate geranyltransferase gene in greater quantities in its midflower than the midflower of Finola, the hemp cultivar. The CBDAS-like paralogs are less abundant in Finola **[see** **Supporting Information—Table S3****]**, despite them being significantly more expressed when compared to PK’s midflower (Table 1). The THCAS-like paralog is expressed in higher levels in the marijuana-type plant PK, and this comparison is significantly different in the three tissues. The roots of PK seem devoid of transcripts of either the CBDAS or THCAS paralog, likely due to the lack of trichomes in this tissue. These results suggest considerable divergence in expression level, especially due to the two order-of-magnitude difference between the expression level of the CBDAS-like paralogs (000395 and 008242) and the THCAS-like paralog (001774).

**Table 1.** Expression for cannabinoid synthase-pathway genes. The expression level for the paralogs related to cannabinoid production varies in both cultivars and tissues. The first column shows each of the paralogs from the PBBK assembly; columns 2, 3 and 4 show the average FPKM (fragments per kilobase of transcript per million fragments mapped), which is a measure of expression level proportional to the number of reads sequenced from that transcript after normalizing for transcript’s length, for transcript levels across runs and for the total yield of the sequencing instrument. Columns 5, 6 and 7 show the significance between the pairwise tissue comparison, and finally column 8 shows the group for each of the paralogs.

				Comparisons			
Paralog	PK midflower (FPKM)	Finola midflower (FPKM)	PK root (FPKM)	PK midflower–Finola midflower	PK midflower–PK root	Finola midflower–PK root	Group
003891	243.5	16.5	0	NS	*P* < 0.05	NS	Olivetolate geranyltransferase
006591	4.39	0.22	0	NS	*P* < 0.01	NS	Unknown cannabinoid synthases
007887	4.383	0.221	0	NS	*P* < 0.0001	NS	
004341	4.38	0.22	0	NS	*P* < 0.01	NS	
002936	4.24	0	0	*P* < 0.01	*P* < 0.01	NS	
005134	0	3.52	0	*P* < 0.01	*P* < 0.01	NS	
000395	0.084	2.516	0	NS	NS	*P* < 0.01	CBDAS-like
008242	0.468	2.75	0	NS	NS	*P* < 0.03	
001774	484.73	1.48	0	*P* < 0.03	*P* < 0.0001	*P* < 0.03	THCAS-like
007396	142.91	6.08	0	*P* < 0.001	*P* < 0.0001	*P* < 0.0001	CBCAS-like
004650	140.94	5.67	0	*P* < 0.003	*P* < 0.0001	*P* < 0.0001	
006705	146.99	6.05	0	*P* < 0.003	*P* < 0.0001	*P* < 0.0001	

## Discussion

In this study, we estimated the CN for the genes encoding enzymes catalysing three of the main reactions of the biochemical pathway that produces cannabinoids (Fig. 1) in the plant *C. sativa*. Although CN variation in some genes involved in cannabinoid production has been previously reported (van Bakel *et al.* 2011; McKernan *et al.* 2015), here we estimate CN variation in multiple steps of the biochemical pathway in 67 *Cannabis* genomes from multiple varieties within the broad-leaf, narrow-leaf and hemp groupings (Lynch *et al.* 2016) using two genome assemblies constructed via complementary technologies.

Our results suggest that synthases for the cannabinoid pathway are highly duplicated and that plants probably use and express the paralogs of these genes differently in specific tissues. Gene CN variation has also been found to be associated with SNP variation and both factors can influence gene expression (Stranger *et al.* 2007). Our results suggest that this is the case for quantitative and qualitative (amount and type) cannabinoid diversity, which seems to be a product of sequence in agreement to previous research (Onofri *et al.* 2015), CN variation (McKernan *et al.* 2015) and expression—after the results presented in this analysis. The effect of CN variation in relation to these mentioned factors that may affect cannabinoid phenotype is an important topic for further study.

### CBDA/THCA synthase family

The lack of *d*N/*d*S value differences and the short genetic distance **[see** **Supporting Information—Table S6****]** suggest that the THCAS/CBDAS gene paralogs arose from a recent duplication event and so have lacked time to accumulate changes. Clusters unique to each of the two assemblies (Fig. 2) suggest that either these clades were selectively lost from the opposing assembly or that there exist lineage-specific paralog combinations. The latter would imply that the acquisition and loss of paralogs is rapid enough to show polymorphism at the cultivar level. Interestingly, all three putative CBDAS paralogs from these two high-THCA marijuana-type assemblies bear premature stop codons (Fig. 2). This finding supports previous research that suggests that marijuana-type cultivars with high-THCA production lack fully functional CBDAS genes (van Bakel *et al.* 2011; Onofri *et al.* 2015; Weiblen *et al.* 2015).

### Gene CN statistics

The difference in CN between hemp and the other two lineages for the three CBDAS-like and the two THCAS-like paralogs (Fig. 3) imply that a whole gene cluster was either lost in most of the hemp cultivars or was duplicated in the marijuana-type (broad-leaf and narrow-leaf) individuals. However, even though the hemp group has the lowest mean and median, for many of these genes it has the widest range in gene CN **[see** **Supporting Information—Table S4****]**, indicating the widest gene CN variation between the three lineages. Copy number for these genes differs little between the broad-leaf and narrow-leaf marijuana types, suggesting similar between-group diversity and higher within-group variation (Fig. 3). Our estimates indicate that some of the analysed individuals from the three different groups could have up to 10 copies of CBDAS/THCAS paralogs **[see** **Supporting Information—Table S3****]**.

### Phenotypic analysis

#### CN vs. chemotype correlation.

There is a positive correlation between accumulation of THC and CN for four of the five paralogs related to CBDA/THCA production, but negative correlation between these paralogs and the accumulation of CBD (Figs 4 and 5; **see** **Supporting Information—Table S5**). This suggests that increasing THCAS gene CN decreases CBDA production possibly due to competition for the mutual precursor, CBGA. Additionally, the THCAS allele from marijuana-type plants appears to be dominant over the THCAS allele from hemp after expression analyses of crossed individuals bearing these alleles, and the CBDAS gene seems to be a better competitor for CBGA even when functional copies of THCAS genes are present (Weiblen *et al.* 2015). This difference in affinity towards CBGA, and in performance from the various genes and alleles, implies significant contributions from both sequence variation and differences in expression of synthase paralogs to differential accumulation of cannabinoids.

The positive correlation between the CN of the paralogs related to CBDA production (000395 and 008242; **see** **Supporting Information—Table S7**) suggests that these paralogs are physically proximal and were possibly copied in tandem (Weiblen *et al.* 2015; Grassa *et al.* 2018). This finding agrees with recent research suggesting that cannabinoid genes are found in close proximity, in tandem repeats, and surrounded by transposable elements (Grassa *et al.* 2018; McKernan *et al.* 2018) which make up between 43 and 65 % of the *Cannabis* genome (Pisupati *et al.* 2018). Both paralogs’ CN correlated with the PK paralog 74778 CN **[see** **Supporting Information—Table S7****]**, and the three paralogs cluster together (Fig. 2), implying that the 74778 paralog in the PK assembly is related to CBDA production. However, the CN of the THCAS-like paralog (001774) is not correlated to the CN from the THCAS-like paralog from the PK assembly (paralog 19603; **see** **Supporting Information—Table S7**) even though they are closely related (Fig. 2). Finally, our BLAST analysis to two other assemblies also shows that these cannabinoid genes are in close proximity **[see** **Supporting Information—Table S8****]**, as reported in their respective publications (Grassa *et al.* 2018; McKernan *et al.* 2018).

Another factor that can affect the correlation between synthase gene CN and THCA and CBDA levels is the presence of truncated genes. High-THCA marijuana cultivars seem to possess a truncated version of the CBDA synthase (van Bakel *et al.* 2011; Onofri *et al.* 2015; Weiblen *et al.* 2015). The presence of the truncated CBDAS paralogs can explain some of the points in Fig. 4 in the bottom right corner where, even though the estimated CN is high (high value on the X-axis), the amount of CBD produced is low (low value on the Y-axis) due to the premature termination and inability to produce the protein. Truncated genes have also been reported for THCA synthases (van Bakel *et al.* 2011; Onofri *et al.* 2015; Weiblen *et al.* 2015); however, we do not see many samples in the bottom right corner with high CN and low THC production (Fig. 5).

It is interesting that the individual hemp-type plants have the lowest mean and median CN for the three CBDAS/THCAS paralogs (Fig. 3; **see** **Supporting Information—Table S4**). We expected hemp types to have a higher mean CN of the two paralogs related to CBDA production, because of their higher production of CBDA compared to marijuana types (de Meijer *et al.* 1992; Rustichelli *et al.* 1998; Mechtler *et al.* 2004; Datwyler and Weiblen 2006). However, hemp individuals have a higher mean for other paralogs from the CBDA/THCA synthase family **[see** **Supporting Information—Table S4****]** such as paralog 005134 which has a negative correlation with the production of THCA but positive for CBDA **[see** **Supporting Information—Table S5****]**. Finally, recent research suggests that CBDA-dominant lineages seem to produce minor cannabinoids which are absent in certain THCA lineages, implying the loss of cannabinoid genes in these highly hybridized THCA-dominant cultivars (Mudge *et al.* 2018). Perhaps these paralogs found in the hemp lineages may be related to these minor cannabinoids, which is subject for further research. Because of the recent aggressive human selection for THCA (Volkow *et al.* 2014), selection for these other genes with yet unknown products is possible.

### Expression analysis

Variation in expression profiles of the THCAS and CBDAS gene paralogs (Table 1) could be another major contributor to measured phenotypic differences among *Cannabis* cultivars, as seen for genes related to stress response in maize (Waters *et al.* 2017). This effect may be augmented by the fact that chemotype assays are generally performed on mature flower masses. Variation in transcription is seen for many of the CBDAS/THCAS paralogs by both tissue and cultivar, suggesting differential use of pathway genes. On the other hand, transcripts from most cannabinoid synthase paralog clades are transcribed in greater quantities by the marijuana cultivar PK in marked contrast to the hemp cultivar Finola (Table 1), implying that marijuana cultivars express more diversity in cannabinoid synthase genes, despite hemp having the widest range in gene CN **[see** **Supporting Information—Table S4****]**. Copy number variation can correlate positively or negatively with gene expression (Stranger *et al.* 2007), which could be the case for THCAS and CBDAS, as may be the particular case for paralog 008242 that has a significant negative correlation with CBDA production. Finally, our results suggest that the enzymes found upstream of the pathway (such as olivetolate geranyltransferase) may play an important role in the production of cannabinoids, which would be regulated by enzymes found in multiple steps of the pathway. However, in order to conclusively make these claims, further studies must include the chemotypes, transcriptomes and genomes of individual plants.

### CN variation and the cannabinoid pathway

The ecological function of cannabinoids is still unknown; however, some suggest that cannabinoids are thought to abate stresses such as UV light or herbivores (Langenheim 1994; Sirikantaramas *et al.* 2005). In other plant species such as potatoes and maize, species-specific secondary metabolites accumulating in glandular trichomes confer resistance to pests and the corresponding synthase genes are found in high CNs (Hardigan *et al.* 2016; Waters *et al.* 2017). This appears to be the case in *Cannabis*. Phytocannabinoid synthesis appears to be genus-specific and accumulation in glandular trichomes could be stress-related (Langenheim 1994; Sirikantaramas *et al.* 2005). Our results suggest that the CBDA/THCA synthase family has recently undergone an expansion. Previous studies have assumed that CBDAS was the ancestral gene and that THCAS arose after duplication and divergence (Onofri *et al.* 2015), but since no other species is known to share this biosynthetic pathway it is not possible to conclusively identify the ancestral state. Our phylogenetic analysis suggests that these cannabinoid genes are specific to *Cannabis.* This is a unsettled topic, however, since there appears to be no remaining truly wild (non-feral) *Cannabis* populations, and even though recent research claims to have identified a homolog of CBDAS in *Humulus lupulus* (Padgitt-Cobb *et al.* 2019), which is *Cannabis*’ closest related extant species, we think this may be a gene that is equally related to all cannabinoids found in *Cannabis* and therefore equally related to CBDAS and to the other cannabinoids.

Regardless, duplication and neofunctionalization of ancestral synthase genes is a likely contributor to chemotype variability. Copy number variants can serve as a mechanism for species-specific expansion in gene families involved in plant stress pathways (Hardigan *et al.* 2016; Waters *et al.* 2017). Additionally, CN variation has been reported in gene families involved in stress response and local adaptation in plants (Hardigan *et al.* 2016; Waters *et al.* 2017), and other organisms (Van de Peer *et al.* 2017), perhaps explaining why all genes in the cannabinoid pathway have been highly duplicated.

The high numbers of paralogs in the CBDAS/THCAS family support the notion that biosynthesis proteins that have fewer internal metabolic pathway connections have a higher potential for gene duplicability (Prachumwat and Li 2006; Yamada and Bork 2009). However, despite both olivetolic acid synthase and olivetolate geranyltransferase operating near the pathway hub, the respective estimated CNs of their paralogs are similar to the CN of CBDA/THCA synthase paralogs (Fig. 1; **see** **Supporting Information—Table S4**). Sequence similarity and physical proximity of extant paralogs in the genome (Weiblen *et al.* 2015; Grassa *et al.* 2018) promotes tandem duplication, again facilitating rapid expansion of the CBDA/THCA synthase family. Human selection since the ancient domestication of this plant has likely played a role, as it did with CN in resistance genes in the plant *Amaranthus palmeri* (Gaines *et al.* 2010) and in the starch digestion gene *Amy2B* during dog domestication (Ollivier *et al.* 2016). Finally, gene CN variation has been associated with SNP variation and both of these factors can influence phenotype expression (Stranger *et al.* 2007).

Our study provides another example of the high association between the CBDA/THCA synthase gene family, which has a very particular relationship, compete for the same precursor molecule (Page and Boubakir 2014; Page and Stout 2017), have a similar chemical structure in their genetic sequence (Brenneisen 2007; Flores-Sanchez and Verpoorte 2008; Onofri *et al.* 2015) and may exemplify ‘sloppy’ enzymes (Auldridge *et al.* 2006; Franco 2011; Chakraborty *et al.* 2013). These ‘sloppy’ enzymes could convert similar substrates (such as CBGA) into a range of slightly different products, such as CBDA, THCA or CBCA (Jones *et al.* 1991).

### Caveats

In addition to the factors previously examined as contributing to the high intrinsic genomic complexity of cannabinoid synthesis pathway regulation, the possible misassembly of both genomes may further confound attempts at precise correlations. For instance, multiple genome assemblies find cannabinoid synthase genes to be clustered in close proximity (Grassa *et al.* 2018; McKernan *et al.* 2018), which is also supported by genetic mapping and inheritance data (Weiblen *et al.* 2015). However, in the PK genome assembly the cannabinoid synthases are found in multiple distinct locations (Laverty *et al.* 2019), which may represent true biological variation or errors in the PK assembly. Nevertheless, all of these studies support the presence of multiple distinct paralogs as members of the cannabinoid synthase gene family. Additionally, the finding of some synthases exclusively in one or the other assembly suggests data gaps in both genomes, although the differences may represent true biological variation due to the high amount of CN variation among the different *Cannabis* varieties. This second hypothesis, suggesting that these differences are true biological variation, is supported by our results presented here. Finally, having only full chemotype data from a single hemp cultivar is a limitation of our study. However, the high CBDA production of this cultivar and our findings of possible deletions in THCA synthase are supported by work which included many hemp genotypes (de Meijer *et al.* 1992; Rustichelli *et al.* 1998; Mechtler *et al.* 2004; Datwyler and Weiblen 2006).

## Conclusions

In this work, we quantify and describe, in multiple ways, the surprisingly high amount of variation in one of the highest revenue-producing biochemical pathways in nature. This gene CN variation and its potential relationship to cannabinoid production has huge medical and agricultural implications. Given that the function of most of these paralogs (Fig. 2) is still unknown, there is potential that some of these genes encode synthases whose products may be of medical importance. Since most medical studies have been performed with the governmentally produced *Cannabis* that has little diversity and potency and does not reflect that produced by the private markets (Vergara *et al.* 2017; Schwabe *et al.* 2019), this work opens the door for more in-depth research, suggesting specific plant lineages deserving of future study. In the agricultural realm, continued work in this area has huge implications for breeding. Because breeders and growers have selected for high levels of THCA (Volkow *et al.* 2014), our results suggest potential ways it would be possible to breed for higher levels of other cannabinoids and related compounds, including those coded by the still-unknown genes (Fig. 2). We hope this study will encourage further research on these genes, particularly as the world moves to legalize this plant.

Returning to our three initial questions: (i) Do lineages differ in number of cannabinoid synthase paralogs? We found that the measured CN of these genes did vary, within and between lineages and possibly within named cultivars given by the differences in CN **[see** **Supporting Information—Table S4****]**. (ii) Does cannabinoid content correlate with the number of respective synthase paralogs by cultivar? We found a positive correlation between the accumulation of specific cannabinoids and the CN of certain synthase paralogs. THCA levels are significantly and positively correlated with the CN of several of these paralogs (Figs 4 and 5; **see** **Supporting Information—Table S5**). Furthermore, the broad-leaf and the narrow-leaf marijuana types each have a higher mean and median for the CNs of genes related to the production of both THCA and CBDA relative to hemp cultivars. However, CBDA levels are negatively correlated with most of the paralogs related to its production, and the hemp cultivars paradoxically exhibit higher CNs for the PK contig 19603 THCAS-like paralog than for CBDAS paralogs **[see** **Supporting Information—Fig. S1**, **Table S5****]**. We found both positive and negative correlations between the production of the other cannabinoids and the CN of some of the paralogs, making it difficult to associate particular cannabinoids with specific paralogs (Figs 3 and 4; **see** **Supporting Information—Fig. S2**). (iii) Do cannabinoid synthase paralogs vary in expression level by tissue and cultivar? We observed differential transcription levels of these genes by tissue in conjunction with cultivar (Table 1) which likely adds to the high complexity of correlating paralog CNs with cannabinoid accumulation.

Finally, our findings motivate a pair of general breeding strategies. To boost production of THCA, select parents with higher CNs of THCAS paralogs, whereas for cultivars with more CBDA, select parents with fewer such paralogs. As cultivars express synthases from multiple points in the pathway differently (Table 1), all of these genes should be considered for breeding purposes. For exclusive production of either THCA or CBDA, cross cultivars bearing only truncated paralogs of the opposing synthase genes.

## Supporting Information

The following additional information is available in the online version of this article—

**Table S1.** Genes from the cannabinoid pathway.

**Table S2.** WGS information.

**Table S3.** Average depth and chemotypes.

**Table S4.** Statistics for differences in copy number (CN) between and within groups, and within repeated strains including modified assemblies.

**Table S5.** Correlations between the estimated copy number (CN) of the 19 different paralogs (including the paralogs from the modified assemblies) and the chemotype for five cannabinoids corrected for relatedness.

**Table S6.** Genetic distance (upper half) and *d*N/*d*S ratio (bottom half) for the 16 CBDAS/THCAS paralogs.

**Table S7.** Correlations between the estimated copy number (CN) of the 19 different paralogs including the paralogs from the modified assemblies corrected for relatedness.

**Table S8.** BLAST results to two newly published assemblies.

**Table S9.** Exons and introns for olivetolic acid and olivetolate geranyltransferase synthases.

**Figure S1.** Estimated copy number (CN) by group for the two of the CBDAS/THCAS paralogs from the Purple Kush (PK) assembly.

**Figure S2.** Correlations between the percent CBDA and the percent THCA and the estimated copy number (CN) for two CBDA/THCA synthase paralogs from the PK assembly.

plz074_suppl_Supplementary_Methods

plz074_suppl_Supplementary_Tables

## Data

The data for this project are available in the dryad repository: https://datadryad.org/stash/share/MsyF2os_zaKN6d9uoDLroX7O0RrW8kT8sPzep7WffLU.

### Sources of Funding

This research was supported by donations to the University of Colorado Foundation gift fund 13401977-Fin8 to N.C.K. at and to the Agricultural Genomics Foundation and publication was funded by the University of Colorado Boulder Libraries Open Access Fund.

### Contributions by the Authors

D.V. analysed the copy number data, wrote the first draft of the manuscript, conceived and lead the project; E.L.H. wrote bioinformatic pipelines for the depth, GLS models and expression analyses, organized the manuscript’s code and made it publicly available; K.G.K. helped with the original bioinformatic code and project conception; R.G., A.T., C.G.C. designed, supervised and provided chemotype data collection; R.M.G., A.T. selected and extracted SMRT-LR template DNA; N.C.K. conceived and directed the project. All authors contributed to statistical analysis and manuscript preparation.

### Conflict of Interest

D.V. is the founder and president of the non-profit organization Agricultural Genomics Foundation, and the sole owner of CGRI, LLC. R.G., A.T., C.G.C. and R.M.G. are employees of Steep Hill, Inc. N.C.K. is a board member of the non-profit organization Agricultural Genomics Foundation.
